# A unified coding strategy for processing faces and voices

**DOI:** 10.1016/j.tics.2013.04.004

**Published:** 2013-06

**Authors:** Galit Yovel, Pascal Belin

**Affiliations:** 1 School of Psychological Sciences and Sagol School of Neuroscience, Tel Aviv University, Tel Aviv, Israel; 2 Institute of Neuroscience and Psychology, University of Glasgow, Glasgow, UK; 3 Département de Psychologie, Université de Montréal, Montréal, Canada; 4 Institut des Neurosciences de La Timone, UMR 7289, CNRS and Université Aix-Marseille, France

**Keywords:** face recognition, voice recognition, neural selectivity, sensory coding, visual cortex, auditory cortex

## Abstract

Both faces and voices are rich in socially-relevant information, which humans are remarkably adept at extracting, including a person's identity, age, gender, affective state, personality, etc. Here, we review accumulating evidence from behavioral, neuropsychological, electrophysiological, and neuroimaging studies which suggest that the cognitive and neural processing mechanisms engaged by perceiving faces or voices are highly similar, despite the very different nature of their sensory input. The similarity between the two mechanisms likely facilitates the multi-modal integration of facial and vocal information during everyday social interactions. These findings emphasize a parsimonious principle of cerebral organization, where similar computational problems in different modalities are solved using similar solutions.

## Similar cognitive and neural representations for faces and voices

Faces and voices are the most socially important stimuli in the visual and auditory domains, respectively. The nature of the sensory input associated with these key social stimuli is very different: reflections of light on the face vs air pressure waves generated by the vocal apparatus. Yet, they both convey very similar types of information about a person, including identity, gender, emotional state, and age. Furthermore, in many cases of social communication faces and voices are processed simultaneously and have been shown to have facilitatory effects on recognition of person information relative to when each is presented alone (for a review, see [1]; Box 1). It is therefore plausible that, despite their very different sensory input, they may generate, at least to some extent, a similar representation. Indeed, recent studies reveal many similarities between their neural and cognitive representations.

Box 1 **Face–voice integration for person information**

Face-voice integration has been primarily studied in the context of speech processing. However, given that faces and voices convey important and similar non-speech information about person identity, it is also important to examine face–voice integration for the processing of identity, emotion, age, and gender. Recent studies have shown that face–voice integration contributes significantly to the extraction of person information [77]. Specifically, cross-modal interaction in the processing of face and voice identity has been shown in studies that presented congruent and incongruent identity [78]. Face–voice integration for gender has been shown even with pure tones extracted from male and female voices, which were not recognized by participants as male or female voices. These pure tones biased perception of an androgynous face to a male or a female face according to the gender of the tone [79]. Integration effects between faces and voices have also been observed for emotional information 80, 81, 82.

Face–voice integration appears very early in life. Several studies have shown that at two months of age, infants begin to exhibit the ability to perceive face–voice correspondences [83]. Interestingly, perceptual narrowing, which has been shown for faces and speech (see main text), has been reported also for face–voice integration. For example, four–six- and eight–ten-month-old infants were presented with consistent and inconsistent face–voice stimuli of monkeys and humans. Whereas the four–six–month-old infants were able to match face-voice stimuli of both humans and monkeys, eight–ten-month-old infants were able to match human but not monkey face–voice stimuli 84, 85. The similar developmental track that is found for faces and voices presented in isolation, as well as for the integration of the two stimuli, is in line with the idea that similar coding mechanisms of unisensory information may underlie successful multisensory integration.

In this review, we highlight the many similarities that have been found between the neural and cognitive mechanisms of face and voice processing in the past few years. We will summarize evidence pertaining to the following five areas: neurophysiological mechanisms; neurocognitive disorders; functional architecture; perceptual coding; and development and experience (Table 1; see Glossary). Because faces have been studied more extensively than voices, we will also highlight several well-established phenomena that have been reported for faces and should be investigated in future studies with voices to further explore their unified coding strategy.

**Table 1:** Face voice similarities

	Face	Voice
**Neural selectivity**		
**Human**		
**Electrophysiology**	N170/M170 [13]	FTPV 14, 17
**Functional MRI**	Face areas in the lateral occipital, mid fusiform and STS 2, 86	Voice areas in the STS [87]
**Hemispheric asymmetry**	Right hemisphere 21, 22	Right hemisphere voice-selectivity [21] (left hemisphere for speech)
**Effects of TMS**	TMS over the OFA selectively impairs performance for faces [18] and selectively increases the face N170 [19]	TMS over the TVA disrupts voice detection [20]
**Monkey**		
**Electrophysiology**	Face-selective cells [23]	Voice-selective cells [26]
**Functional MRI**	Face-selective brain areas 4, 23	Voice-selective brain areas [25]
**Selective recognition deficits**		
	Developmental and acquired prosopagnosia 29, 30	Developmental and acquired phonagnosia 31, 34
**Perceptual Coding**		
**Norm-based coding** (Box 2)	Relative to an averaged face 40, 42, 88	Relative to an averaged voice 39, 41
**Distinctiveness effect**	Better recognition for distinctive faces [37]	Better recognition for distinctive voices [89]
**Perceptual aftereffects to anti-faces/voices** (Box 2)	Largest for matched vs non-matched anti-faces [88]	Largest for matched vs non-matched anti-voices [39]
**Attractiveness** (Box 3)	Averaged face is more attractive [90]	Averaged voice is more attractive [91]
**Development and experience**		
**Early preference**	Preference for upright faces 24 hours after birth [43]	Fetuses and young infants discriminate voices from other auditory stimuli 45, 46
**Neural correlates**	Face-selective ERPs appear at three–six months [50]	Voice areas emerge between three and seven months 52, 53
**Perceptual narrowing**	Broad abilities for cross species face recognition at four–six months are tuned by experience in eight–ten-month-old infants [54]	Broad abilities for phoneme discrimination at four–six months are tuned by experience in eight–ten-month-old infants [56]
**Effects of experience in adulthood**	Other race effect [60]	Language familiarity effect [57] and own-race bias [59]

The many similarities that exist between the neural and cognitive representation of faces and voices suggest a unifying coding mechanism that has evolved to represent the very rich and diverse information that these unique classes of visual and auditory stimuli convey about a person. More generally, these findings suggest that the brain may employ similar principles for processing stimuli that convey similar types of information not only within the same modality, but also across different modalities.

## Neurophysiological mechanisms

**Figure 1:**
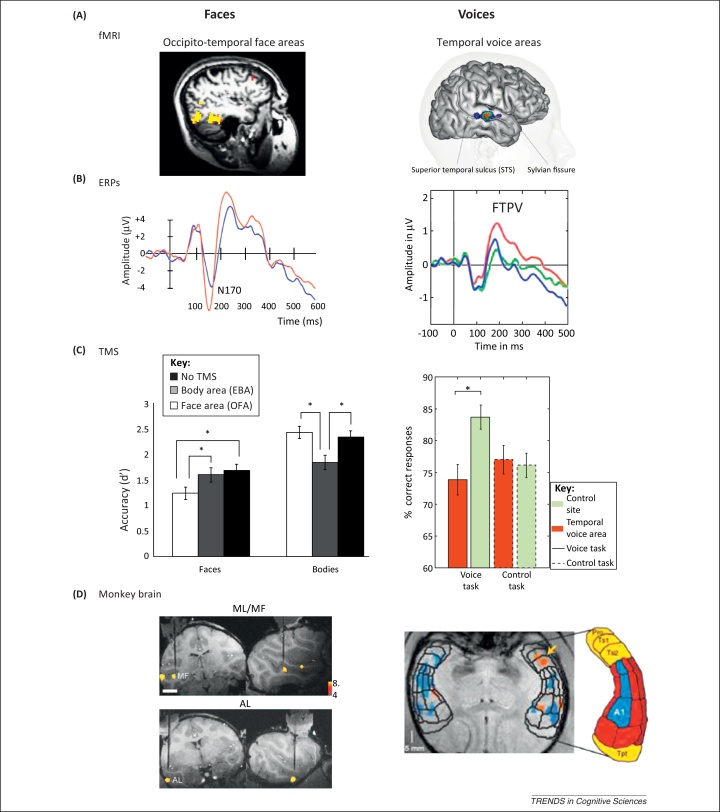
Face and voice-selective neural responses. **(A)** Left: face-selective areas revealed with functional MRI (fMRI) are shown in the occipital temporal cortex. Right: the voice-selective areas are found in superior temporal sulcus and gyrus. **(B)** Left: faces elicit greater event related potential (ERP) amplitudes than non-faces 170 ms after stimulus onset – N170 in occipito-temporal electrodes (red line – faces). Right: voices elicit greater amplitudes that non-voice sounds 200 ms after stimulus onset in fronto-temporal electrodes (red line – voices). Reproduced, with permission, from [14]. **(C)** Left: transcranial magnetic stimulation (TMS) to the occipital face area selectively disrupts face but not body discrimination. Adapted from [18]. Right: TMS to the temporal voice area selectively disrupts voice/nonvoice discrimination. Reproduced, with permission, from [20]. **(D)** Left: face-selective areas found in the superior temporal sulcus of the macaque brain. Reproduced, with permission, from [23]. Right: voice-selective areas were found in the superior temporal plane of the macaque brain. Reproduced, with permission, from [25].

Faces and voices have both been shown to elicit highly selective neural responses in the human brain (Figure 1A–C). Faces have been typically compared to non-face objects, such as houses or chairs. Voices are usually compared to different categories of non-vocal sounds, such as environmental or mechanical sounds. Functional MRI (fMRI) studies reveal much stronger responses to faces than any other non-face stimuli in at least three occipital temporal areas: the occipital face area (OFA) in the lateral occipital cortex, the fusiform face area (FFA) in the mid fusiform gyrus, and a face area in the posterior superior temporal sulcus (STS–FA) 2, 3 (Figure 1A, left). Recent studies also reveal more anterior face-selective responses in the anterior temporal lobe and the prefrontal cortex [4]. Voice-selective cortical mechanisms do also exist: fMRI studies have identified several regions along the middle and anterior STS and superior temporal gyrus (STG) that show a greater response to vocal sounds (regardless of whether they carry intelligible speech or not [5]) than to non-vocal sounds 6, 7, 8: these areas were named the ‘temporal voice areas’ (TVA) (Figure 1A, right). Voice-sensitive responses have also been observed in other areas, including the insula and prefrontal cortex 9, 10, 11.

Consistent with neuroimaging findings, electroencephalography (EEG) and magneto-encephalography (MEG) studies show face- and voice-selective evoked responses. Faces elicit a component of much larger in amplitude than non-face stimuli 170 ms after stimulus onset – the face-selective N170/M170 12, 13 (Figure 1B, left). A voice-selective electrophysiological component at a latency comparable to that of the N170, termed the ‘fronto-temporal positivity to voice’ (FTPV), has been also recently reported in EEG 14, 15, 16 (Figure 1B, right) and MEG [17] studies approximately 200 ms after sound onset. Finally, transcranial magnetic stimulation (TMS) of fMRI-defined face-selective areas indicates a causal and specific role for the occipital face area in face discrimination (Figure 1C, left) and in the generation of the face-selective N170 response 18, 19. Similarly, TMS over the TVA has been shown to disrupt voice detection [20] (Figure 1C, right).

Finally, one prominent and well-established feature of the face-processing mechanism is its right hemisphere asymmetry, which has been manifested both in neural and behavioral measures 21, 22. Whereas speech processing is lateralized to the left hemisphere, voice recognition, similar to faces, elicits neural responses that are right lateralized [21].

Face- and voice-selective neural responses are not limited to the human brain, but have also been observed in the macaque brain. Face neurons are commonly found in the superior temporal sulcus and the inferotemporal cortex [23]. Furthermore, functional MRI studies reveal a network of face-selective areas primarily in the upper and lower banks of the superior temporal sulcus [4] that share at least some anatomical and functional similarities with the human face areas [24] (Figure 1D, left). Similarly, monkey fMRI studies revealed voice-selective areas [25] in the superior temporal plane that prefer species-specific vocalizations over other vocalizations and sounds (Figure 1D, right). These voice-selective areas have been shown to contain voice-selective neurons [26]. The presence of face- and voice-dedicated mechanisms in the macaque brain indicates that these face and voice areas did not just emerge recently in humans along with the emergence of language and high-level social functioning skills: they were probably already present in the last common ancestor of macaques and humans some 30 million years ago. This highlights the importance of these stimuli for basic social functioning throughout primate evolution.

In summary, neurophysiological and neuroimaging findings convincingly show that both faces and voices elicit a highly selective neural response. This highlights not only their social importance, but also the fact that the unique nature of their representation requires mechanisms that are different from those used for the processing of any other visual and auditory stimuli. Moreover, this similarity in their neural representations is consistent with other similar principles used for the processing of auditory and visual stimuli, such as the tonotopic and retinotopic representations in primary auditory and visual cortex, respectively, or the separate mechanisms for ‘where’ and ‘what’ information that have been reported both in visual [27] and auditory [28] systems.

## Neurocognitive disorders

Consistent with the strong neural selectivity that is discussed above for faces and voices, neuropsychological studies have reported selective impairments in face or voice recognition, in the face of otherwise intact visual or auditory functions, respectively. Selective deficits in face recognition abilities (i.e., prosopagnosia) were reported over 50 years ago in brain-damaged patients following a lesion in the occipital temporal cortex, usually over the right hemisphere [29]. More recently, similar deficits were found in individuals that show no specific brain lesion, but suffer from life-long prosopagnosia, known as developmental/congenital prosopagnosia [30]. Prosopagnosic individuals seem to show intact recognition of objects, but exhibit severe difficulties in recognizing familiar faces including their close relatives and friends. Regarding voices, the existence of patients with selective impairments in speech comprehension has long been established (e.g., Wernicke's aphasia). More similar to prosopagnosia, a small number of ‘phonagnosic’ patients have been identified with impairments in speaker discrimination or recognition, even though other aspects of auditory perception were normal 31, 32, 33. Only one case of ‘developmental phonagnosia’ – the selective inability to recognize speakers by their voice in the absence of any evident cerebral impairment – has been reported so far [34]. It is possible that the lack of additional developmental phonagnosia cases may not reflect an absence of such cases, but the inability of individuals who suffer from this deficit to acknowledge their deficit, as was the case with developmental prosopagnosia for many years. Furthermore, a lack of standardized tests for phonagnosia also impedes its reliable diagnosis.

## Functional architecture

As mentioned above both faces and voices convey similar information about a person, including gender, emotional state, identity, and age. The idea that the functional architecture underlying face and voice processing could be organized following comparable principles has been discussed before and therefore will only briefly mentioned here 1, 35. A neurocognitive architecture described by Bruce and Young [36] has been suggested to also apply to voices [1]: briefly, after a stage of cortical processing common to all stimuli of their particular sensory modality, faces and voices are selectively processed in a further ‘structural encoding’ stage, probably represented by areas such as the FFA and TVA, respectively. Then, in each modality, the three main types of information carried by both faces and voices – identity, affect, speech – are processed along functional pathways which, although they interact with one another during normal functioning, can be selectively activated/impaired.

## Perceptual coding

One of the most influential models of face processing is the ‘face space model’ [37], which posits that face identity can be represented as locations in a multidimensional space. The dimensions of this space correspond to information used to discriminate faces, whereas the distance that separates representations reflects the degree of similarity between faces. This similarity-based framework accounts for a range of face-recognition phenomena, such as the face inversion effect, effects of distinctiveness and caricaturing, and the other race effect [37]. Furthermore, single unit recording studies in the macaque show neuronal tuning profiles that are consistent with such similarity-based representations [38]. Current evidence suggests that all faces are coded relative to a prototypical, average face, which lies at the origins of the multidimensional face space (Box 2).

Box 2 **Multidimensional face and voice spaces**

The idea that faces and voices are coded relative to a norm has received its main support from studies that employed behavioral adaptation paradigms. Adaptation entails exposure to a stimulus for a relatively long duration of a few seconds. This long exposure generates perceptual aftereffects during the presentation of a subsequent stimulus, such that the representation of the adapted stimulus becomes weaker and its ‘opposite’ becomes stronger. For example, after long exposure to the color green, a white screen appears red because of opponent red–green color coding in the retina.

Aftereffects, which were originally used to detect the properties of low-level sensory stimuli, such as color and motion, have been later found also for face gender, identity, and age 92, 93, 94. For example, long exposure to a female face generates a stronger male perception in a 50%/50% female–male morphed face. Face aftereffects have also been useful as tests of the properties of the multi-dimensional face space. In particular, according to the norm-based coding hypothesis, all faces are coded as a function of their distance relative to an average face that lies in the origin. Findings showed greater aftereffects for two stimuli that are located in opposite sides of the average face (a face and an anti-face) than two faces that are not on the axis that goes through the origin where the average face resides (see Figure IA–C) 38, 40. These findings provide strong support for the idea that faces are coded in a norm-based manner relative to an average face.

**Figure I:**
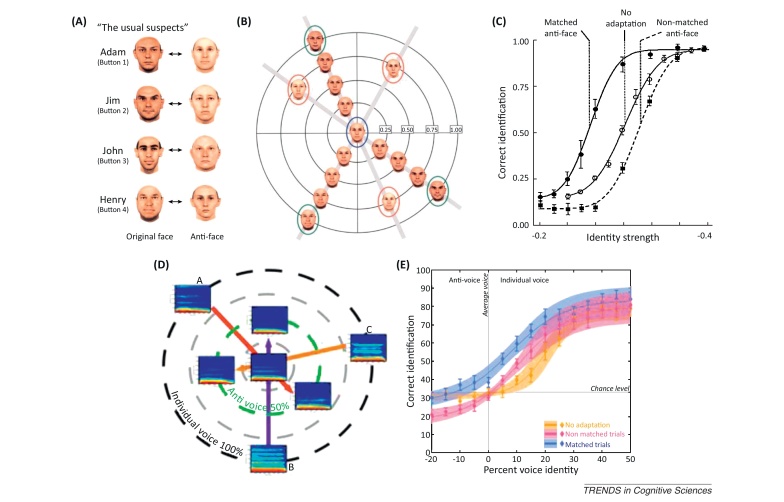
Perceptual aftereffects of ‘anti-face’ and ‘anti-voice’ adaptation. **(A–C)** Anti-face adaptation. **(A)** Four face identities used in a recognition task (left column) and their corresponding ‘anti-face’ versions (right column); note the very different identity precepts associated with a face and its anti-face; yet, they are related in that averaging them together results in the average face. **(B)** Stimuli used in recognition tasks represented in a theoretical multidimensional space centered on the average face (blue circle). Green circles indicate learned identities. Red circles indicate anti-faces. **(C)** Psychophysical labeling functions obtained as a function of increased identity strength at baseline (no adaptation: continuous line, open symbols) and after adaptation (closed symbols) with matched (continuous line) and non-matched (dashed line) anti-face adaptors. Note the greater aftereffects induced by matched anti-face adaptors and the strong identity percept associated with the otherwise identity neutral average face (identity strength 0) after adaptation with matched anti-faces. Reproduced, with permission, from [40]. **(D–E)** Anti-voice adaptation. **(D)** Three voice stimuli (brief syllables represented by their spectrogram) shown in a theoretical multidimensional space, with an averaged voice in its center, and with their corresponding anti-voice stimuli (on the green circle). **(E)** Psychophysical labeling function obtained as a function of increased identity strength at baseline (no adaptation: orange symbols) and after adaptation with matched (blue symbols) and non-matched (pink symbols) anti-voice adaptors. Note, as for faces, the greater aftereffects induced by adaptation with matched anti-voice adaptors. Reproduced, with permission, from [39].

Interestingly, recent aftereffect studies with voices reveal similar effects for voice information such as gender [95], identity 96, 97, and emotion [98]. Voice aftereffects also provide evidence for norm-based coding of voice identity: identity aftereffects induced by ‘anti-voice’ adaptors are greater in magnitude than those induced by non-opposite adaptors [39]. As for faces, the average voice, normally perceived as identity-free, becomes tainted with the identity of the opposite to the anti-voice adaptor (Figure ID,E), even though voice and anti-voice are not perceived as related in identity.

Recent studies have uncovered very similar phenomena for the coding of voice identity. Voices from different speakers can be represented as points in a multidimensional space (Box 2). Similar to faces, a prototypical voice stimulus can be generated by averaging together a large number of different voices of the same gender. A particular role of this prototypical voice has been shown via perceptual aftereffects induced by adaptation with ‘anti-voices’ [39] in an experimental paradigm directly adopted from face experiments [40]. Cerebral activity in the TVA has recently been shown to vary as a function of a voice's acoustical distance to the prototypical voice [41] – i.e., “norm-based coding”. This is analogous to results from the fusiform face area which showed increase in signal with increased distance from the mean face 38, 42.

## Development and experience

Given the importance of face and voice recognition for intact social functioning and the specific computations that are needed to extract the rich information that they convey, it may not be surprising that processing mechanisms for faces and voices appear very early in development. A specific preference for upright faces in infants has been found during the first 24 hours after birth [43]. These findings suggest that face processing mechanisms may be innate and that early on face-like figures attract attention more than other non-face stimuli [44]. Similarly, there is clear evidence that very young infants – even fetuses – can discriminate voices from other auditory stimuli and can recognize their mother's voice 45, 46. By the age of three months, infants also prefer listening to human voices than to vocalizations from other species [47].

Early evidence for neural selective responses to faces or voices also exists. For faces, one positron emission tomography (PET) study with two-month-old infants has shown face-selective responses (faces > diodes) in the lateral occipital and the fusiform gyrus. Although the choice of control stimuli was not ideal, these areas may correspond to the adult OFA and FFA [48]. Event related potential (ERP) studies with three-month-old infants reveal face-selective components – the N290 and N400 49, 50. These components emerge later than the adult N170 and spread over a longer time range. Thus, face-selective neural mechanisms may exist at early infancy, but are further sharpened during development. With respect to information carried by voices, the contrast of fMRI measures of activity for speech vs reversed speech already shows an adult-like left-lateralized pattern at three months [51]. Evidence of greater response to vocal vs non-vocal sounds seems to emerge slightly later, between three and seven months, as shown by near-infrared spectroscopy (NIRS) and fMRI 52, 53. Notably, newborns already exhibit a neural signature for voice identity recognition [46].

Evidence for early, possibly innate, existence of face and voice selective mechanisms does not imply that their development is not influenced by experience. Perceptual narrowing during infancy has been reported for both face and speech stimuli. In particular, at six months of age infants can recognize both monkey and human faces, but the former ability declines by nine months, when face recognition becomes better for human faces 54, 55. Similar perceptual narrowing has been reported for speech [56]. The language spoken in one's cultural group is an obvious such influence of experience, with evidence for cerebral mechanisms tuned to the specific set of phonemes of the maternal language within the first year after birth (see Box 1 for perceptual narrowing of face–voice integration).

Non-linguistic aspects of voice perception, such as speaker recognition, also seem to be susceptible to environmental influence: it is well established that listeners recognize speakers of their own or a familiar language better than speakers of an unfamiliar language, the language familiarity effect 57, 58 and there is partial evidence for a potential effect of race on voice recognition [59]. This phenomenon may parallel the well-established ‘other race effect’ – humans’ poor ability to recognize faces of other races (e.g., Asian faces by Caucasian observers and vice versa) [60], which results from the little contact with faces of other races. Taken together, evidence suggests that mechanisms selective for the processing of faces and voices appear very early in development and may even be innate. These mechanisms are widely tuned to all types of face and voice/speech stimuli early on, but narrow down already by nine months of age and remain narrowly tuned to the type of faces and voices one has experience with also in adulthood.

## Unexplored face–voice similarities

Whereas ample evidence already exists for the similar coding of faces and voices, many phenomena that have been discovered in the extensive study of faces in the past 50 years still await testing with voice stimuli. Crucially, several behavioral phenomena have suggested a special status for faces compared to non-face objects, but no such effects are known for vocal stimuli. These would include a voice correlate of the face inversion effect [61] and/or the contrast reversal effects (stimulus manipulations that result in a disproportionately large recognition deficit relative to non-face stimuli [62]. Another hallmark of face processing is its holistic representation [63], which is manifested by interactive, rather than independent, representation of the face parts. Testing whether these well-established face-specific effects have their counterparts in the auditory domain may be a fruitful avenue of research. For instance, studies using a gating paradigm or examining the effects of transformation such as time reversal or frequency reversal (or ‘rotation’ [64]) on different stimuli could potentially highlight effects specific to vocal sounds 65, 66.

Other phenomena that have been extensively studied with faces are the different representations of familiar and unfamiliar faces 67, 68. For example, the representation of familiar faces is more tolerant to stimulus manipulations such as viewpoint or lighting changes relative to unfamiliar faces. Also, faces are detected more rapidly than other objects in visual scenes and search arrays [69] and have been shown to capture attention relative to other objects [70]. It is still unknown whether voices have a similar privileged status relative to other sounds.

Finally, faces automatically elicit social inferences about the personality of the individual 71, 72. Interestingly, it has been shown that these inferences can be clustered into two main independent inferences, trustworthiness and dominance 71, 72. Evidence for a similar two-dimensional space that maps onto trustworthiness and dominance has also been suggested for voices [73]. Future studies will determine whether trustworthiness and dominance are correlated with voice expression and voice gender, respectively, as was shown for faces [74].

## Concluding remarks

Visual and auditory signals have very different physical properties and are processed by separate neural substrates. Nevertheless, the visual and auditory pathways do employ some similar mechanisms, including the retinotopic and tonotopic representations seen in early sensory cortices and a separation to ‘what’ and ‘where’ pathways in both vision and audition 27, 75. In this review, we have shown that the two systems also apply very similar computational operations to the processing of their categories of overriding ecological importance, faces and voices. This is manifested in category neural selectivity to faces and voices that was found both in human and macaque brains, selective cognitive impairments, and early appearance in development. Furthermore, similar norm-based coding schemes for identity and attractiveness (Box 3) and separate, but interactive pathways for identity expression and speech have been demonstrated (Table 1). These similarities, as well as others that should be explored in futures studies (Box 4), are likely to contribute to effective face–voice integration (Box 1), which has been shown to result in recognition that exceeds the sum of each of the stimuli alone.

Box 3 **Are averaged faces and voices more attractive?**

It has been shown for over a century that ‘averaged faces’ generated by averaging together a number of different faces are highly attractive 90, 99 (Figure IA). Evolutionary theory proposes that averaged faces are more attractive because they contain features that are indicators of fitness in natural faces (the ‘good genes’ account): symmetry, averageness, texture smoothness 100, 101. A more cognitive explanation of this phenomenon is in terms of similarity to the internal prototype, which results in easier to process, more pleasant stimuli (‘perceptual fluency’) [102].

**Figure I:**
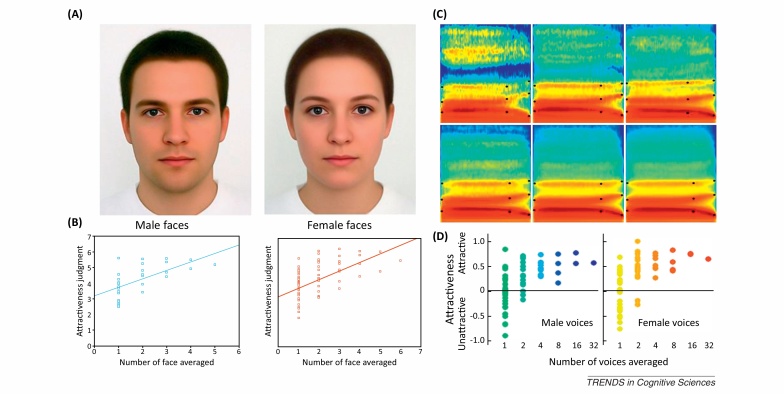
Face and voice attractiveness judgments as a function of averaging. **(A)** Face composites generated by averaging 32 male faces (left) and 64 female faces (right). **(B)** Attractiveness ratings as a function of number of face averaged. Note the steady increase in attractiveness ratings with increasing number of averaged faces, for both male (left) and female (right) faces. Reproduced, with permission, from [106]. **(C)** Spectrograms of voice composites generated by averaging an increasing number of voices of the same gender (different speakers uttering the syllable ‘had’). **(D)** Attractiveness ratings as a function of number of voices averaged. Note the steady increase in attractiveness ratings with increasing number of averaged voices, for both male (left) and female (right) voices. Reproduced, with permission, from [91].

Both the good genes and perceptual fluency accounts predict that a similar phenomenon should be observed for voices. Bruckert *et al.* [91] used morphing (Figure IB) to generate voice composites made of an increasing number of voices and observed, as predicted by face studies, a significant increase in attractiveness ratings. Two main acoustical parameters were highlighted, both analogous to those shown to influence face attractiveness: distance-to-mean (acoustical similarity with the population average); and ‘texture smoothness’ (i.e., amount of spectro-temporal irregularities) [91].

Note that for both faces and voices, averageness appears to be one factor among many that influence the attractiveness percept. Other factors, such as sexual dimorphism, are also known to contribute to both face and voice attractiveness in a complex, context-dependent manner 103, 104, 105.

Box 4 **Outstanding questions**

• Is the perceptual and cerebral processing of unfamiliar voices different in nature from that of highly familiar voices, as has been demonstrated for faces?
• Is there ‘holistic’ processing in representing voice? Can ‘voice inversion’ or ‘voice composite’ effects be observed?
• Is the threshold for voice detection lower than for other sound categories? Do voices capture more attention than other auditory stimuli?
• Are there any neural/perceptual effects that are specific to voices that should be studied with faces?
• Is the neural system that mediates face processing more extensive than the neural system that mediates voice processing?

Note that this review has largely focused on the similarity between faces and voices. However, these two stimuli also differ in important ways. Importantly, human face recognition abilities surpass the ability to recognize people by voices [e.g., 76]. This may not be surprising given the fact that humans are highly visual species. Whether this difference reflects a more complex organization of the face network with, for example, more areas (as the data available on voice areas in the human or macaque brain suggest) or a less informative signal to start with (1-dimensional sound frequency vs 2-dimensional visual spatial), or both, remains to be established.

## Glossary

Adaptation: prolonged exposure to a stimulus modulates neural and behavioral responses to the stimulus.
Aftereffects: prolonged exposure to a stimulus distorts perception of a new stimulus that follows it in time, in a direction opposite to the first stimulus.
Face inversion effect: decline in recognition for upside-down relative to upright stimuli is larger for faces than any other non-face objects.
Fusiform face area (FFA): a face-selective area that is found in the human fusiform gyrus. Other occipito-temporal face-selective areas are found in the lateral occipital and superior temporal sulcus.
Other race effect: recognition of faces of other races (e.g., Caucasian faces by Asian observers) is poor relative to own race faces (e.g., Asian faces by Asian observers).
Retinotopy: the visual field is mapped topographically onto neurons in the retina and early visual cortex such that adjacent neurons encode nearby regions in the visual field.
Temporal voice areas (TVA): voice-selective areas found in the middle and anterior parts of the human superior temporal sulcus/gyrus bilaterally, with a right-hemispheric asymmetry.
Tonotopy: sounds of different frequency are organized topographically in the ascending auditory pathways and early auditory cortex, such that nearby neurons are tuned to nearby frequencies.
Perceptual narrowing: discrimination abilities among sensory stimuli in young infants (three–six months old) are reduced in older infants (nine months old) for stimuli for which they have no perceptual experience with (e.g., monkey faces, monkey face–voice integration).
Phonoagnosia: selective deficit in voice recognition. Most cases reported have been due to brain damage in the right temporo-parietal junction. A single case of developmental phonagnosia (i.e., with no apparent brain damage) has been described.
Prosopagnosia: selective deficit in face recognition, which appears in two forms, acquired and developmental. Acquired prosopagnosia results from brain damage, usually in the right temporal lobe. Developmental cases suffer from a life-long deficit with no apparent brain damage.
Voice inversion: voice stimuli can be reversed in time or inverted (‘or rotated’) in frequency, inducing recognition deficits.
